# A Case Report on 13q12.3 Microdeletion Syndrome Caused by *HMGB1* Haploinsufficiency

**DOI:** 10.1155/crig/1912620

**Published:** 2024-11-27

**Authors:** Ting Wen, Brian J. Shayota, Lauren Wallace, Coumarane Mani, Neal Davis, Jian Zhao

**Affiliations:** ^1^Department of Pathology, University of Utah School of Medicine, Salt Lake City, Utah 84108, USA; ^2^ARUP Laboratories, Salt Lake City, Utah 84108, USA; ^3^Pathology and Laboratory Medicine, Henry Ford Hospital, Detroit, Michigan 48202, USA; ^4^Division of Medical Genetics, Department of Pediatrics, University of Utah School of Medicine, Salt Lake City, Utah 84112, USA; ^5^Intermountain Hillcrest Pediatrics, Murray, Utah 84107, USA

**Keywords:** 13q12.3 microdeletion syndrome, atopy, developmental delay, *HMGB1* deletion

## Abstract

Heterozygous microdeletions at 13q12.3 are associated with a rare genetic disorder, 13q12.3 microdeletion syndrome, characterized by intellectual disability, microcephaly, development delay, facial dysmorphisms, atopy, and obesity. Reported 13q12.3 microdeletions vary in size and typically encompass multiple genes. Previous studies have defined a minimal overlap region of 13q12.3 microdeletions and suggested that most of the phenotype associated with the 13q12.3 microdeletion syndrome could be attributed to the loss of the high mobility group box 1 (*HMGB1)* gene within the overlap region. Here, we report a pediatric patient who had typical phenotypic features of 13q12.3 microdeletion syndrome, including motor and moderate speech developmental delays, microcephaly, and severe atopy, along with anxiety and aggressive behaviors. Trio-based microarray analysis identified a 62-kb apparently *de novo* heterozygous deletion at 13q12.3 in the proband, fully encompassing all coding exons of the *HMGB1* gene yet not affecting any other neighboring genes. This case report presents a rare *HMGB1* single-gene deletion in a patient with classic features of 13q12.3 microdeletion syndrome, allowing a better delineation of clinical phenotypes associated with the loss of *HMGB1*. Our findings, together with previous reports, strongly support the pathogenic role of *HMGB1* haploinsufficiency in the 13q12.3 microdeletion syndrome.

## 1. Introduction

13q12.3 microdeletion syndrome is an emerging genetic disorder that has been described in a small number of patients with overlapping features including developmental delay, intellectual disability, facial dysmorphisms, eczema/atopic dermatitis, microcephaly, and obesity [1–5]. To date, the underlying gene causing this syndrome has not been fully understood. Previously, the minimal overlap region shared among patients with 13q12.3 microdeletions was proposed to include three protein-coding genes (*KATNAL1*, *UBE2L5*, and high mobility group box 1 [*HMGB1*]) and two long noncoding RNAs (*LINC00426* and *LINC01058*) [1, 2]. Within this minimal region, the pathogenic role of *HMGB1* in the 13q12.3 microdeletion syndrome has been further implicated in a recent study reporting the association of heterozygous loss-of-function sequence variants in *HMGB1* with developmental delay and microcephaly [3].

The *HMGB1* gene encodes an evolutionarily conserved DNA-binding factor involved in various biologically important processes, including cell signaling, chromatin looping, DNA repair, and the inflammatory response [6]. Mechanistically, the *HMGB1* protein regulates transcription factor binding and chromatin remodeling. Functionally, its immunological regulatory role is highlighted by noted secretion of *HMGB1* from damaged cells and activated immune cells, thereby functioning as a cytokine and inducing an inflammatory response [7]. *HMGB1* protein also has an impact on the development of the central nervous system by promoting neurite outgrowth and cell migration and enhancing forebrain development in the rodent system [8]. The functional roles of *HMGB1* in immune and central nervous systems support the notion that the loss of the *HMGB1* gene could be responsible for at least part, if not all, of the phenotype associated with 13q12.3 microdeletion syndrome.

In addition to the 13q12.3 microdeletion syndrome, heterozygous pathogenic variants in *HMGB1* have been associated with brachyphalangy, polydactyly, and tibial aplasia/hypoplasia syndrome due to two different disease-causing mechanisms [5, 9]. Although partial deletions or loss-of-function variants predicted to result in haploinsufficiency of *HMGB1* have been suggested to contribute to the phenotype observed in the 13q12.3 microdeletion syndrome [3, 5], additional evidence, including cases of isolated *HMGB1* full-gene deletions, is required to further support this hypothesis.

Herein, we report an apparently *de novo HMGB1* single-gene deletion detected by chromosomal microarray in a patient with highly overlapped clinical features of the 13q12.3 microdeletion syndrome. This finding supports the notion that most of the known manifestations of the 13q12.3 microdeletion syndrome can be attributed to haploinsufficiency of *HMGB1*.

## 2. Materials and Methods

### 2.1. Patient Consent and Ethical Statement

Written informed consent was obtained from the patient's family for the publication of clinical information and photographs. This study was performed in accordance with protocols approved through the University of Utah Institutional Review Board.

### 2.2. Microarray Analysis

Genomic DNA was extracted from peripheral blood of the proband and buccal swab of his parents. Cytogenomic SNP microarray was performed using the CytoScan HD Suite (Thermo Fisher Scientific) according to validated protocols within the Genomic Microarray Laboratory at ARUP Laboratories. The microarray data were analyzed for copy number variants (CNVs, gains, and losses) as well as copy-neutral alterations (regions of homozygosity) using the Chromosome Analysis Suite (ChAS) software (version 4.4.1). Parental relationships were confirmed using the Mendelian inheritance error (MIE) tool in ChAS. CNV classification was performed in accordance with recommendations by the American College of Medical Genetics and Genomics (ACMG) 2020 technical standards [10]. Genomic coordinates correspond to the Genome Reference Consortium human genome build 37/human genome issue 19 (GRCh37/hg19).

## 3. Results

### 3.1. Clinical Description

**Figure 1:**
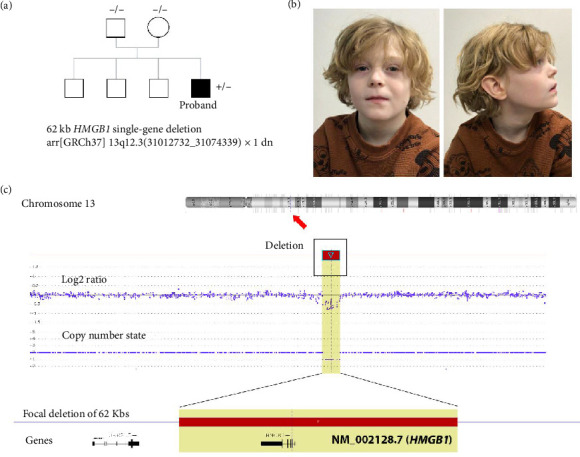
Clinical features and genomic profile of the proband with *HMGB1* single-gene deletion. (a) Pedigree depicting the apparently *de novo HMGB1* single-gene deletion with no significant family history. (b) Front and side photographs of the proband at the age of 9 years. (c) Microarray signal pattern showing the *HMGB1* heterozygous deletion at 13q12.3, with a red arrow (macroscopic) and yellow highlight (microscopic) indicating the deleted region.

The patient is a 9-year-old male presenting with mild motor and moderate speech developmental delays, speech apraxia, microcephaly (as low as 1.6th percentile, −2.13 SD), anxiety, aggressive behaviors, and severe atopy including urticaria, atopic dermatitis, eosinophilia, and multiple food allergies. He also experienced significant infantile constipation that has since resolved with age and has a fascination playing with and being in water. Other growth parameters were low-normal, with a height in the 11th percentile and weight in the fifth percentile. Physical examination was remarkable for general hypopigmentation of the hair and skin relative to the biological parents and siblings, upper eyelid fullness, posteriorly rotated ears, long philtrum, and thin vermillion upper lip (Figure 1). Family history is insignificant, with both parents and three elder brothers all reportedly in good health. Except for microarray, no other genetic testing was performed on this patient.

### 3.2. Microarray Findings

An approximately 62-kb heterozygous deletion within 13q12.3 was detected by a microarray in the patient (GRCh37/hg19 coordinate chr13: 31012732–31074339) (Figure 1). This deletion removes every coding exon of all known *HMGB1* transcripts, resulting in complete loss of one functional copy of the *HMGB1* gene. This deletion exclusively affects the *HMGB1* gene and does not include any other genes in the neighboring region. Subsequent parental testing by microarray did not detect this deletion, suggesting the *de novo* status of the *HMGB1* deletion in the patient. No other abnormal findings were detected in this patient by microarray. Based on available information, the *HMGB1* deletion was considered clinically significant and was classified as likely pathogenic.

## 4. Discussion

In this study, we identified an apparently *de novo HMGB1* single-gene deletion in a boy with clinical features resembling those documented in patients with the 13q12.3 microdeletion syndrome. This finding supports the notion that haploinsufficiency of *HMGB1* plays a critical role in the pathogenesis of the 13q12.3 microdeletion syndrome.

Over the past decade, the 13q12.3 microdeletion syndrome has been described in approximately a dozen patients [1–5, 11]. Common clinical features reported in the literature include developmental delay, language delay, moderate intellectual disability, unique facial dysmorphisms, eczema/atopic dermatitis, microcephaly, and obesity. Of these features, our patient exhibits developmental delay, language delay, microcephaly, and an atopic phenotype including food allergy. In addition, our patient displays some of the reported facial features associated with the 13q12.3 microdeletion syndrome, including upper eyelid fullness, posteriorly rotated ears, and a thin vermillion upper lip. Although obesity is not noted in our patient, it may be influenced by a number of other factors, including the possibility of it being masked by the dietary management of the patient's food allergy. Taken together, our patient's phenotype closely aligns with most of the described features associated with the 13q12.3 microdeletion syndrome.

**Figure 2:**
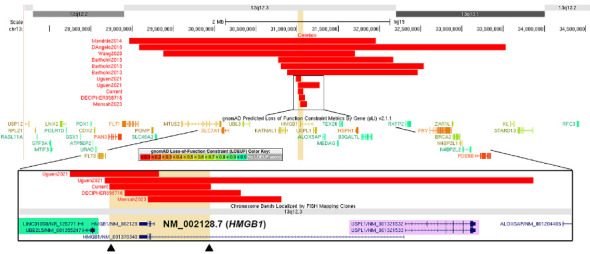
A schematic literature review of 13q12.3 microdeletions. Reported 13q12.3 microdeletions are centered around the minimal overlap region. A zoomed view below illustrates the nature of the current *HMGB1* single-gene deletion, with the break points (black triangles) of the current deletion (highlighted in yellow) shown. The current deletion includes every coding exon of all known *HMGB1* transcripts, fully encompassing the clinically relevant *HMGB1* transcript NM_002128.7 reported in Uguen et al. and Mensah et al.'s studies. This deletion does not affect the neighboring genes *LINC01058* and *UBE2L5* (highlighted in green) and *USPL1* (highlighted in purple). This figure was generated using the UCSC Genome Browser.

Reported 13q12.3 microdeletions in the literature vary in size from 54 kb to 3.4 Mb (Figure 2). Among them, the minimal overlap region has been narrowed down to a focal locus encompassing *HMGB1* and a few other genes, namely, *KATNAL1*, *UBE2L5*, *LINC00426*, and *LINC01058* [1–3, 5]. The smallest 54 kb deletion reported so far by Uguen et al. fully encompasses all coding exons of *HMGB1* but also affects neighboring genes *UBE2L5* and *LINC01058*. Mensah et al. reported a small deletion that involves the first three coding exons but not the entire *HMGB1* gene. At this time, it remains unclear how deletions of the neighboring genes of *HMGB1* may impact the phenotype. Therefore, additional evidence of the isolated *HMGB1* full-gene deletion is essential to establish the pathogenic role of monoallelic *HMGB1* loss in the 13q12.3 microdeletion syndrome. The deletion identified in our patient exclusively affects *HMGB1* and includes every coding exon of all known *HMGB1* transcripts, which is expected to result in haploinsufficiency of *HMGB1* excluding potential confounds of neighboring genes. To the best of our knowledge, this is the first report of an isolated *HMGB1* full-gene deletion in a patient with classic clinical features of the 13q12.3 microdeletion syndrome. Notably, DECIPHER has also documented an isolated *HMGB1* full-gene deletion, classified as uncertain pathogenicity, in a child with global developmental delay and language impairment (DECIPHER case number 398716, Figure 2), consistent with the finding in our patient.

In a rare scenario, an apparently *de novo* interstitial deletion present in a child could be inherited from one of the parents who is a carrier of a balanced insertional translocation [12]. We showed that the *HMGB1* deletion present in the proband was not detected in his parents by a microarray, with parental relationship confirmed by the MIE tool. However, due to the small size of this deletion, further analysis by metaphase FISH could not be performed to rule out the possibility of a balanced insertional translocation in one of the parents. Therefore, the *HMGB1* deletion present in the proband was presumed *de novo*.

In addition to deletions, *HMGB1* frameshift variants with a putative loss-of-function effect have been identified in patients with clinical features of the 13q12.3 microdeletion syndrome [3, 13], supporting the etiological contribution of *HMGB1* haploinsufficiency to the pathogenesis of the 13q12.3 microdeletion syndrome. However, the loss-of-function effect of these *HMGB1* frameshift variants requires further confirmation through functional studies. It is important to note that frameshift variants located in the last exon of *HMGB1* are associated with a severe malformation syndrome (BPTAS; OMIM 609945), characterized by brachyphalangy, polydactyly, and tibial aplasia/hypoplasia [5, 9]. Notably, BPTAS and 13q12.3 microdeletion syndrome are two clinically distinct disorders caused by different disease-causing mechanisms involving *HMGB1*, although limb anomalies such as finger camptodactyly and clinodactyly have been observed in some patients with deletions or loss-of-function variants of *HMGB1*. BPTAS is attributed to the gain-of-function effect of frameshift variants that replace the acidic carboxy-terminal tail of *HMGB1* with an arginine-rich basic tail, whereas the 13q12.3 microdeletion syndrome is likely due to the loss-of-function of *HMGB1*.

The role of *HMGB1* in allergic reactions/atopy is established in the research [6]. Of interest, atopic features (such as food allergy and atopic dermatitis) represent key manifestations present in the majority of patients with the 13q12.3 microdeletion syndrome [1, 2, 4], which could help establishing clinical diagnosis and facilitate gene dosage ontology studies of the key player *HMGB1*.

In conclusion, we report a heterozygous deletion of *HMGB1* in a patient exhibiting clinical features of the 13q12.3 microdeletion syndrome. This is the first documented case of an isolated *HMGB1* full-gene deletion. Our findings provide direct evidence that *HMGB1* haploinsufficiency is the causal mechanism underlying most of the characteristic features of the 13q12.3 microdeletion syndrome. To date, the *HMGB1* gene has no known human disease association in Online Mendelian Inheritance in Man (OMIM) nor has it been evaluated for a dosage effect by ClinGen. Our results could assist in the establishment of a clinical diagnosis for patients carrying deletions or loss-of-function variants involving *HMGB1*.

## Data Availability

The data that support the findings of this study are available from the corresponding author upon reasonable request.
